# Implementing a Fascia Iliaca Compartment Block Curriculum in an Emergency Medicine Residency Program

**DOI:** 10.7759/cureus.58472

**Published:** 2024-04-17

**Authors:** Jonathan H Brewer, Jordan Rupp, Jeremy S Boyd

**Affiliations:** 1 Emergency Medicine, Vanderbilt University Medical Center, Nashville, USA

**Keywords:** pain management, emergency medicine, resident education, hip fracture, nerve block, regional anesthesia

## Abstract

With pain being commonly stated as a reason for presentation to the emergency department (ED) and the advent of the opioid crisis in the United States, regional anesthesia has been gaining prominence as an alternative treatment for acute pain in emergency medicine. However, to this date, there is no widely agreed-upon and standardized training regimen for regional anesthesia in emergency medicine residency programs. In this paper, we set out to define elements of competency for a residency program in a large academic tertiary center and to create a protocol for resident training that could be easily replicated, with a secondary goal of increasing the frequency of nerve blocks in the ED. We also aimed to discuss a curriculum that has been shown to improve resident comfortability with the fascia iliaca compartment block (FICB). This led to a substantial increase in nerve blocks performed in the ED. However, we also demonstrate a loss of retention at six months, indicating that further curriculum refinements will be needed to promote longitudinal retention of knowledge.

## Introduction

Pain is the most common reason for emergency department (ED) visits [1]. While emergency physicians are expertly trained to handle a myriad range of complaints, acute pain has been a major challenge in the field of emergency medicine. Many patients feel that their pain is not adequately controlled and that the effect of analgesia is limited during their stay in the ED [2]. Opioid overdoses have resulted in over 80,000 deaths in the United States in 2021 alone [3-4], but the available non-opioid analgesics are often inadequate; hence, many emergency physicians may feel that their arsenal is limited for treating acute pain.

However, emergency physicians are excellent proceduralists. Leung et al. have demonstrated that emergency physicians are competent in ultrasound-guided needle guidance for venous cannulation. In addition, when properly trained in a simulation setting, emergency physicians have demonstrated increased success and decreased complications with ultrasound-guided procedures in comparison with landmark-guided procedures [5-6].

The fascia iliaca compartment block (FICB) is a well-established nerve block; it was first described by Dalens et al. in 1989 [7]. By injecting local anesthetic just beneath the fascia iliaca, the nerve block provides a significant level of analgesia for injuries to the hip and femur, such as femoral neck and peri-trochanteric femur fractures. Additionally, it is relatively easy to be performed by non-anesthesiologists [8] and has been shown to have efficacy in the hands of paramedics, resident physicians, and nurses [9-11]. There are no absolute contraindications except for overlying cellulitis, and it has a better safety profile than femoral blocks or three-in-one blocks [12]. Many patients with hip fractures who present with significant pain are at risk of delirium, altered mental status, and other high-risk events. It is imperative to provide adequate analgesia to these patients, and FICB serves as an opioid-sparing solution to this issue [13-14].

While regional anesthesia is commonly taught during anesthesia residency, it is still a fairly novel technique in the emergency medicine community. Currently, there is no widely utilized standardized curriculum for teaching ultrasound-guided regional anesthesia (UGRA) within emergency medicine residencies and even ultrasound fellowships [15]. In this study, we primarily aimed to define competency in the residency for the FICB with a secondary objective of assessing the number of FICBs performed after initial training.

## Materials and methods

This study was an IRB-approved, prospective cohort review of emergency medicine residents at Vanderbilt University Medical Center (VUMC); a level-one trauma center in Nashville, Tennessee. It included resident emergency medicine physicians. Individuals with varying levels of experience between their first and third year at this job were included so that the sample was properly representative of the entire program. The cohort also included residents who completed an ultrasound rotation during their intern year. Our project endeavored to teach an extremely effective and beneficial modality of pain control to emergency medicine residents at a large, tertiary academic referral center. This initiative employed a combination of simulation-based training along with standardized patients and didactics discussing both the basics of regional anesthesia along with focused training, reviewing the FICB to better serve our patients in the ED.

To conduct this study, Mindray (Mahwah, NJ) M9 and Fujifilm Sonosite (Bothell, WA) PX machines were utilized along with B. Braun Medical (Bethlehem, PA), Ultraplex 360 single shot block needles for both simulation and on-shift training. Images were archived using the Q-path (Telexy Healthcare, Everett, WA) server/system. Images were not only stored but interpreted, and rated on their validity of interpretation by emergency medicine physicians who were fellowship-trained ultrasound providers, as follows: true-positive, true-negative, false-positive, and false-negative. All studies submitted with completed worksheets underwent review to ensure quality standards were maintained.

At the outset, study participants were administered a RedCap (Nashville, TN)-based pre-survey to evaluate their baseline level of knowledge with regard to the basics of regional anesthesia, dosing of local anesthetics, and the FICB (Table 1).

**Table 1:** FICB pretest PGY: postgraduate year; FICB: fascia iliaca compartment block; LAST: local anesthetic systemic toxicity

Questions	Answers
Participant name	
Participant year	PGY-1
	PGY-2
	PGY-3
Which probe is primarily used for the fascia iliaca nerve block	Curvilinear
	Phased-array
	High-frequency linear
	Endocavitary
What is required for monitoring a patient's condition during high-volume nerve blocks? Check all of the applicable boxes	Pulse oximeter
	End-Ttdal CO_2_ monitoring
	Cardiac leads
	Blood-pressure monitoring
What is proper patient positioning during a fascia iliaca compartment block?	The patient sitting at a 45-degree angle
	Left lateral decubitus
	The patient sitting at a 90-degree angle
	Supine
	Prone
Select complications that may occur from nerve blocks	Local anesthetic systemic toxicity
	Intraneural injection
	Hemorrhage
	Intravascular injection
	Injection site infection
What is the maximum dose for bupivicaine 0.5% w/o epi?	2.5 mg/kg
	3 mg/kg
	4.5 mg/kg
	6 mg/kg
What is the maximum dose for ropivicaine 0.5% w/o epi?	2 mg/kg
	3 mg/kg
	4.5 mg/kg
	6 mg/kg
What is the maximum dose for lidocaine 1% w/ epi?	2 mg/kg
	3 mg/kg
	4.5 mg/kg
	7 mg/kg
What is the usual initial sign of local anesthetic systemic toxicity?	Seizures
	Perioral numbness or tingling
	Cardiac arrest
	Loss of consciousness
What is the treatment for LAST? What is the dose?	Intralipid 10%; bolus 1 mL/kg followed by a 0.25 mL/kg/min infusion
	Intralipid 20%; bolus 1.5 mL/kg followed by a 0.25 mL/kg/min infusion
	Intralipid 10%; bolus 1.5 mL/kg followed by a 0.25 mL/kg/min infusion
	Intralipid 20%; bolus 1 mL/kg followed by a 0.25 mL/kg/min infusion
The nerve target for the fascia iliaca nerve block is between the __ and __ muscles	Iliopsoas muscle and fascia iliaca
	Sartorius muscle and fascia iliaca
	Sartorius muscle and fascia lata
	Iliopsoas muscle and fascia lata
An infrainguinal approach to the fascia iliaca nerve block should be expected to anesthetize which nerves? Check all applicable boxes	Femoral nerve
	Lateral femoral cutaneous nerve
	Obturator nerve
	Saphenous nerve
What are the contraindications to a fascia iliaca block? Check all applicable boxes	CHF
	Uncontrolled diabetes
	Overlying infection
	Hypertension
	Previous femoral bypass surgery
Describe in your own words the distribution of a fascia iliaca nerve block	

**Figure 1:**
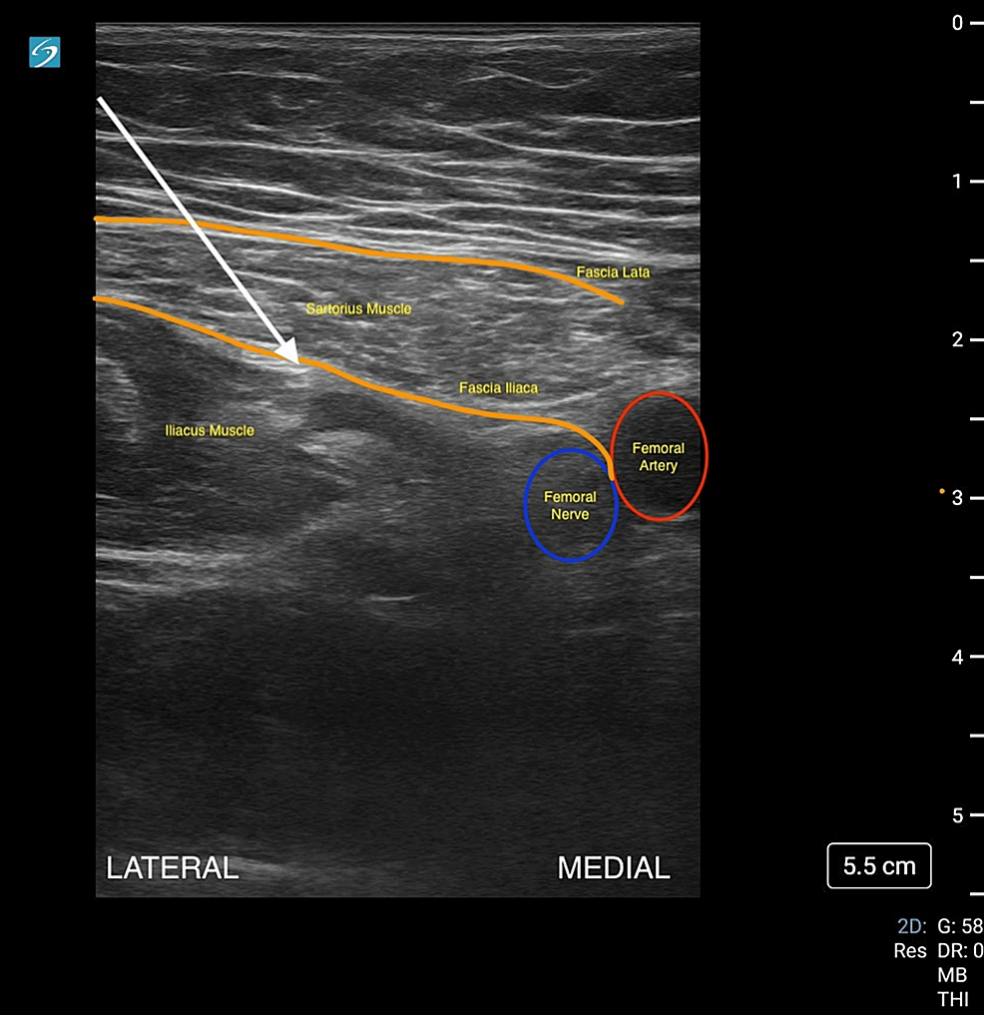
Infrainguinal approach to the fascia iliaca compartment block

This survey was linked to a study ID so that pre- and post-intervention responses could be matched anonymously. Participants then underwent a 30-minute didactic lecture regarding the aforementioned topics along with real-time practice over needle guidance and anatomical recognition delivered by ultrasound fellowship-trained emergency physicians and standardized patients. This lecture included didactics about the usage and safety profiles of various local anesthetics, anatomy for the infrainguinal approach to the FICB, and pre- and post-assessments for ensuring a successful block. The infrainguinal approach (Figure 1) was initially selected as the anatomy is similar to other femoral procedures that emergency medicine residents may be comfortable with, such as a femoral arterial line or central venous catheter. Afterward, residents were allowed to conduct the infrainguinal approach to the fascia iliaca block in their clinical work supervised by emergency medicine attendings for certain conditions that are mentioned within the departmental Fascia Iliaca Block Protocol, such as hip fractures.

Our protocol includes 15 mL of ropivacaine 0.5% without epinephrine plus 15 mL of 0.9% sterile normal saline. The patient was then observed with cardiopulmonary monitoring for a minimum of 30 minutes with a repeat pain assessment after the observed time. After six months, a repeat survey was administered to the residents with identical questions to the pre-survey to assess knowledge retention along with a repeat session where residents demonstrated competency with anatomical recognition and in-plane needle guidance using phantom simulators and standardized patients. This session was assessed by the same ultrasound faculty member who was responsible for initially teaching the block to ensure standardization. This fellowship-trained faculty member then filled out a survey where residents were graded along a scale of 0-2 with 0 correlating to a failing grade and 2 corresponding to "exceeds expectations." The final question on the evaluation comprised a scale that ranged from “I would NOT trust them to I would trust them to complete the exam at the bedside by themselves and I would review the images” (Table 2).

**Table 2:** FICB instructor evaluation PGY: postgraduate year; FICB: fascia iliaca compartment block; LAST: local anesthetic systemic toxicity

Questions	Answers
Participant name	
Participant year	PGY-1, PGY-2, PGY-3
Able to describe the distribution of effect from this block	0, 1, 2
Able to describe their material setup (including anesthetic choice/amount) and patient positioning	0, 1, 2
Can point out the anatomy of the block (sartorius m., iliacus m., fascia lata, fascia iliaca, femoral nerve and vessels)	0, 1, 2
Can describe their target and what they should see with injection (unzippering of fascia iliaca from the iliacus muscle	0, 1, 2
Appropriate probe selection	0, 1, 2
Appropriate settings (gain, depth, frequency)	0, 1, 2
Appropriate patient and probe positioning	0, 1, 2
Appropriate adjunct techniques to improve image quality, i.e., patient breathing, probe placement	0, 1, 2
Name the indications for FICB	0, 1, 2
Name the contraindications for FICB	0, 1, 2
Discuss the risks of FICB	0, 1, 2
Can the student describe the symptoms of LAST toxicity?	0, 1, 2
Can the student describe the treatment for LAST toxicity?	0, 1, 2
If you were on shift with the resident this afternoon, how much would you trust them to perform the above nerve blocks?	I would NOT trust them
	I would trust them to perform the exam if I was at the bedside for the entire exam
	I would trust them to begin the exam at the bedside until I could join them at the bedside
	I would trust them to complete the exam at the bedside by themselves and I would review the images

A retrospective review of the Q-path server was also conducted at the end of the six months by ultrasound faculty post-intervention to analyze the total number of FICBs and nerve blocks performed in the ED.

## Results

For the primary outcome of this study, the residents’ level of procedural competency in performing the infrainguinal FICB was assessed both at the time of the educational intervention and six months afterward. In this setting, the goal was to maintain retention of skills while also analyzing the aggregate number of FICBs performed in the ED during this period as a secondary outcome.

Overall, 20 residents participated in this study: eight postgraduate year (PGY)-1 residents, six PGY-2 residents, and six PGY-3 residents; the cohort was evenly distributed with a slight skew towards the PGY-1 year. Of the 20 residents who participated in the study, none received a rating of “I would not trust this resident to perform this procedure” even while under supervision. In addition, no resident received a failing grade (defined as less than 70% on the post-test). This was a marked improvement from the pre-intervention assessment where only 20% were able to delineate the target for a FICB (p=0.0002; 95% CI: 0.56-0.94) on a standardized patient and the pre-test assessment. All of them were able to delineate the correct probe positioning and ultrasound settings as well as describe various ways to improve image acquisition.

While there was a pattern of greater success among the PGY-2 and PGY-3 years in terms of anatomical recognition skills, description of block distribution, and description of FICB risk, everyone was able to describe the symptoms of local anesthetic systemic toxicity (LAST), which was deemed to be a critical action. In addition, residents excelled in their needle visualization and guidance that was tested on a phantom model. However, 81.3% of residents (n=13) received the overall grade of “I would trust them to perform the exam if I was bedside for the entire exam.” Only 12.5% (n=2) of residents received the grade of “I would trust them to complete the procedure by themselves;” meaning that they received top marks on every component that was assessed, from anatomical recognition to technical needle guidance.

The number of FICBs performed in our department by trainees increased, substantially which was probably due to an increased interest in learning and performing nerve blocks. While this was only a marginal increase (12 FICBs vs. two in the previous year), the overall number of total nerve blocks performed in our emergency department increased from 19 in the 2021-2022 academic year to over 100 in 2022-2023.

## Discussion

The data from this study suggests that while the infrainguinal approach to the FICB can be easily taught, retention of this knowledge can be hard if the nerve block is not routinely performed. Residents who sought opportunities to conduct the block retained knowledge better than ones who did not. The two residents with the highest scores also completed an additional advanced ultrasound selective in which regional anesthesia was a component, which may have contributed to their additional competency. However, almost all residents were able to retain knowledge about LAST toxicity and performed well in anatomical recognition along with in-plane needle guidance. Our leading theory as to why they might have uniformly done well is as follows: since this block is similar to the approach for an ultrasound-guided femoral arterial line, the anatomy is well known to the emergency medicine resident.

For our secondary analysis, while we noticed an increase in the number of FICBs and nerve blocks overall in the ED, various factors still limited the overall potential. In the future, emergency medicine faculty education will need to focus on making residents feel comfortable supervising this procedure when an ultrasound faculty member is not available. This curriculum was successfully implemented. Further interventions, such as the use of a simulator, may lead to improved competence and confidence. While there were limitations to this study, it did create a renewed interest among the trainees, which we feel may lead to an increased motivation to perform nerve blocks for the acute treatment of pain at our center and conduct further studies down the road. Overall, this is an easy-to-perform procedure that can provide immense pain relief to patients and should be considered as part of acute pain education in emergency medicine residency programs.

Regarding limitations, much of the current literature focuses on individuals within the anesthesia specialty. There is a scarcity of literature focused on emergency physicians even though this segment could have practice-changing implications if adequate measures are implemented appropriately. Additionally, there are limitations concerning time within the ED that may lead to a decrease in the number of fascia iliaca blocks performed. Depending on volumes within the emergency department and also the procedural experience of supervising physicians, the number of blocks performed could be limited. Furthermore, the power of this study was low due to the number of available participants and the fact that the two best-performing residents had chosen a special ultrasound-based elective in which regional anesthesia was a component.

Our study primarily focused on residents and only 20 out of the 30 total members were available for both sessions of the curriculum. In addition, at the time of the completion of the study, all members of the PGY-1 year had completed their ultrasound rotation where needle guidance is taught as a component of ultrasound-guided intravenous cannulation. This group was also taught by ultrasound fellowship-trained physicians, but any faculty member may supervise the group. While attending physicians are ultimately required to supervise every procedure within the ED, some may feel uncomfortable with this procedure until further training is provided. Finally, the definition of competence for this study was based on a survey that was created by and assessed by a single assessor, which provides consistency but may also introduce an element of bias.

## Conclusions

FICB is an effective modality of pain control, but a single brief educational workshop was not adequate to gain sustained FICB procedural competence at six months for emergency medicine residents. After the educational intervention, there was an increase in the number of FICBs and total nerve blocks performed in the ED. This study demonstrates that while initial intervention has led to an increase in knowledge and interest, further interventions will be needed to ensure that this knowledge is retained.
